# Surface Instability of Bilayer Hydrogel Subjected to Both Compression and Solvent Absorption

**DOI:** 10.3390/polym10060624

**Published:** 2018-06-06

**Authors:** Zhiheng Zhou, Ying Li, Tian Fu Guo, Xu Guo, Shan Tang

**Affiliations:** 1 College of Aerospace Engineering, Chongqing University, Chongqing 400017, China; zhihengzhou@cqu.edu.cn; 2 Department of Mechanical Engineering and Institute of Materials Science, University of Connecticut, Storrs, CT 06269, USA; yingli@engr.uconn.edu; 3 Institute of High Performance Computing, A*STAR, Singapore 138632, Singapore; guotf@ihpc.a-star.edu.sg; 4 State Key Laboratory of Structural Analysis for Industrial Equipment, International Research Center for Computational Mechanics, Department of Engineering Mechanics, Dalian University of Technology, Dalian 116024, China

**Keywords:** hydrogel, hierarchical wrinkles, surface instability, finite element simulation

## Abstract

The bilayered structure of hard thin film on soft substrate can lose stability and form specific patterns, such as wrinkles or creases, on the surface, induced by external stimuli. For bilayer hydrogels, the surface morphology caused by the instability is usually controlled by the solvent-induced swelling/shrinking and mechanical force. Here, two important issues on the instability of bilayer hydrogels, which were not considered in the previous studies, are focused on in this study. First, the upper layer of a hydrogel is not necessarily too thin. Thus we investigated how the thickness of the upper layer can affect the surface morphology of bilayer hydrogels under compression through both finite element (FE) simulation and theoretical analysis. Second, a hydrogel can absorb water molecules before the mechanical compression. The effect of the pre-absorption of water before the mechanical compression was studied through FE simulations and theoretical analysis. Our results show that when the thickness of the upper layer is very large, surface wrinkles can exist without transforming into period doublings. The pre-absorption of the water can result in folds or unexpected hierarchical wrinkles, which can be realized in experiments through further efforts.

## 1. Introduction

Bilayered soft structures are ubiquitous and widely used in many engineering applications [1], such as in sensors [2,3,4,5], in microfluidic devices [6,7], in responsive coatings [8], in smart adhesives [9], and in the control of cellular behaviors [10,11]. These structures typically consist of a thin metallic [12,13], polymeric [14,15], gel [16], or silicate film [17] bonded to a softer elastomer substrate (e.g., poly (polydimethylsiloxane)—PDMS), hydrogel, or polyelectrolyte gel. The controlled size, order, and morphology of surface patterns can be used to realize many functionalities [18,19]. For example, the control of surface patterns allows the regulation and tuning of transport in microfluidic channels [20,21], chemical adhesion [22,23], wetting [24,25], and optical function [18].

Examples of such surface patterns include wrinkles [26,27], creases [28,29,30], folds [31], ripples [32,33], two-dimensional labyrinths, and herringbone patterns [34,35]. These surface patterns are usually detected as defects prior and should be avoided in engineering applications. They are usually related to the natural phenomena of elastic buckling instability under mechanical compression. When a thin film on soft substrate is compressed further beyond the onset of the buckling, new deformation modes may also appear. The patterns of surface instability are summarized in [36,37]. The onset of both buckling and post-buckling are strongly dependent on the modulus ratio between the thin film and substrate, the material property, stress states of the substrate, initial curvature of the surface, and so forth. The buckling instability [31,38,39] can be induced by other stimuli different from the mechanical force, such as ionic strength [40], temperature [41], light [42], solvent composition [16], and electric fields [43,44].

A large amount of literature reporting how to analyze the buckling instability of bilayered soft structures exists. Both linear instability and nonlinear instability analyses are carried out. The effects of the modulus ratio between the thin film and the substrate on the behaviors of surface instability are discussed and dated back to Allen’s monograph [45] and the recent works of [31,46,47]. The stress state of the substrate also plays a key role. A pre-stretched substrate can result in different behaviors of outward or inward deflection, leading to new post-wrinkling modes. For instance, Cao and Hutchinson [31] predicted the emergence of localized ridges as a different type of post-wrinkling bifurcation with large substrate pretension through numerical modeling, which was verified by a few experiments [48]. Auguste et al. [49] studied the post-wrinkling bifurcations on a pre-stretched substrate (both tension and compression) through the combination of experiments and simulations. The pre-stretched substrate not only showed substantial shifts in the critical strain for the onset of post-wrinkling bifurcation, but also exhibited qualitatively different modes of surface instability, such as tripling. Shao et al. [50] analyzed the curvature-induced hierarchical wrinkling patterns in soft bilayers. These theoretical analyses are limited to elastomeric soft solids such as PDMS and silicone rubber. Generally speaking, analyzing the instability of bilayer hydrogels is similar to analyzing the elastomeric soft solids because the actuating stimuli, such as temperature, light, solvent composition, and electric field, to trigger the buckling instability finally can be transferred to the mechanical compression. However, transferring the actuating stimuli to mechanical compression is not always straightforward, particularly when both the mechanical compression and solvent absorption or release occur at the same time. To the best of our knowledge, the surface instability of bilayer hydrogels, subjected to solvent adsorption and mechanical compression simultaneously, has not been explored before. In addition, in previous studies, it is usually assumed that the upper layer is thin compared with the substrate. This may not be the case in many engineering applications. Our horizon also needs to be expanded to consider the case with a thicker upper layer, which may trigger the new instability patterns and can be harnessed in the future applications.

In this work, we combine theoretical analyses, numerical simulations, and experimental studies to understand the surface instability of bilayer hydrogels under both compression and pre-absorption of water. A robust theoretical and numerical framework to identify critical conditions for the onset of surface instability of the bilayered hydrogels under swelling and the combined pre-swelling and compression is built. A detailed constitutive model for hydrogels has been calibrated through extensive experimental data and was adopted to study the transition of surface patterns systematically by varying the material and geometric properties of the bilayer hydrogel. The surface instability of the bilayer hydrogel was found to be governed by both the initial volume fraction of polymer chains, the height ratio, and the pre-absorption of water. Through extensive theoretical analysis and finite element (FE) simulations, a phase diagram is presented, which can guide the experimentalists to realize these different surface patterns.

## 2. Experimental Setup

**Figure 1:**
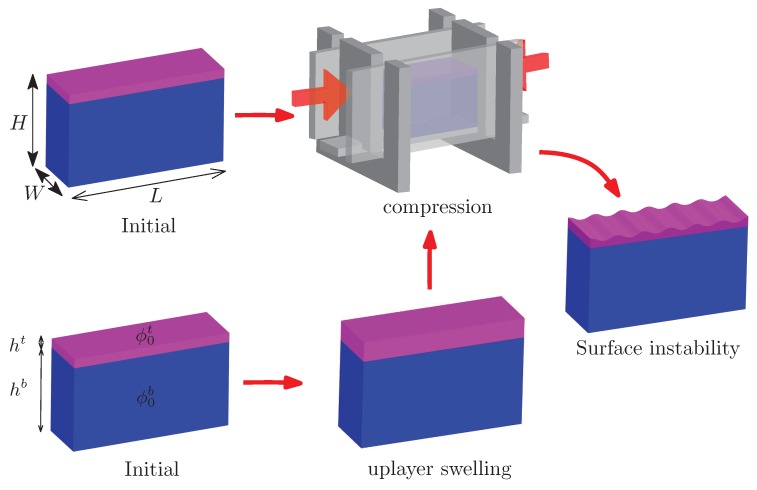
Schematics for two routes to realize the surface instability for the bilayer hydrogel discussed in the present work: (1) only mechanical compression after the fabrication of sample; (2) mechanical compression after the pre-absorption of water in the top layer.

For the experimental setup, an in-house loading apparatus was designed in which a cuboid specimen of a bilayer hydrogel with length *L*, height *H*, and width *W* can be placed, as shown in Figure 1. This loading apparatus has been discussed in our previous works [29]. For all the specimens used in present study, L=4.5 cm, H=2 cm, and W=1 cm. The size and geometry of the specimen can affect the surface instability of the experimental results. It is discussed in [29] that the dimension can control the surface pattern of soft solids. The present results can be reproduced in the neighborhood of the length–height and length–width ratios used in the present study.

This study first fabricated a bilayer hydrogel with different initial volume fractions of water in an in-house glass box. The thickness of the top layer was around 1/17 of the substrate thickness. The fabricated specimen was then placed into the loading apparatus for uniaxial compression. The compressive loading was imposed manually such that the loading rate was extremely low, and the application of compressive loading was accomplished by a pair of screw-driven plates placed width-wise to the specimen. Two glass plates were placed length-wise to the specimen and were held fixed to impose plane strain conditions exactly. Common washing liquids were applied to the contact surfaces of the plates and specimen to reduce the friction between them. Washing liquids are effective to reduce the friction, which has been shown in our previous work [29]. The imposed compressive strain could then be easily measured by the length of the specimen during deformation. The current methodology for compressive experiments has been fully discussed in our previous works [29].

**Figure 2:**
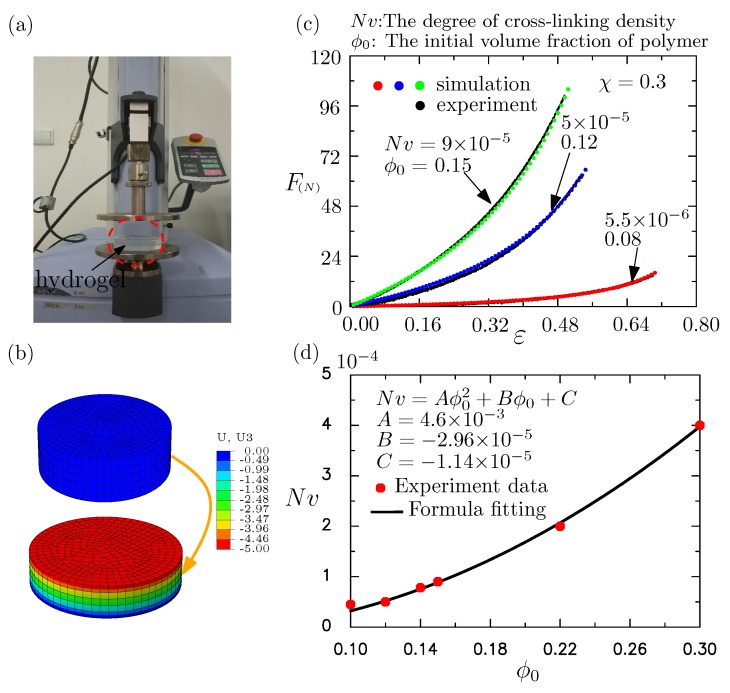
(**a**) The mechanical test machine and the cylindrical specimen through which we obtained the force–strain curves for hydrogels (reproduced from Ref. [51] with permission from The Royal Society of Chemistry.) (**b**) The finite element (FE) mesh used to calibrate the material parameters of the hydrogel. (**c**) The force–strain curves of experimental results fitted by the proposed hydrogel model, where χ is taken to be 0.3. (**d**) The fitting formula of Nv (a measure of the degree of cross-linking or cross-link density of the polymer network) as a function of initial volume fraction of polymer chain $\phi_{0}$ on the basis of the experimental measurements, which is used to describe the material behaviors of both upper and lower layers of hydrogel.

To calibrate the material constants involved in the proposed constitutive model in FE (finite element) simulations, the hyperelastic properties of these hydrogels were measured through uniaxial compression experiments. The uniaxial compression experiments were conducted by loading equipment (SHIMADZU EZ-LX, SHIMADZU, Kyoto, Japan). A photograph of the machine setup is given in Figure 2a, along with the magnified views of the compression fixture. Cylindrical specimens, each of radius 37.5 mm and height 20 mm, of homogeneous hydrogels with various water contents were fabricated and subjected to uniaxial compression tests as shown in Figure 2a. The initial volume fraction of water could be measured because the water content is known during the fabricating process. Hydrogels with different initial volume fractions of polymer $\left(\phi_{0}=0.08,0.1,0.12,0.14,0.15,0.22,\;\mathrm{and}\;0.30\right)$ were fabricated to test the proposed constitutive model, where we denote $\phi_{0}$ as the initial volume fraction of polymer and $1-\phi_{0}$ as the initial volume fraction of water molecules. On the basis of these experiments, it is clear that the initial volume fraction of the water could determine the modulus of these hydrogels. Consistent with our intuition, a larger initial volume fraction of the water means a softer modulus of the hydrogel.

## 3. Numerical Analysis

### 3.1. Material Models

Hydrogels are usually modeled as a hyper-elastic material [52,53,54], whose constitutive response can be described by a free-energy density function. The free-energy density proposed by Hong et al. [55,56] is an attractive model that is dependent on the deformation gradient $F_{ij}$ and chemical potential increment Δμ. However, the free-energy density adopted in their original works takes the dry, solvent-free polymeric network as the reference state. In reality, hydrogels contain some or even large amounts of water molecules and are never dry (such as the samples we fabricated in our experiments). Thus, to reflect this initial physical state of the hydrogel, it is important to take this swollen state of the hydrogel (with water volume fraction $1-\phi_{0}$) as the undeformed reference state. The free-energy density can be written as

(1) $$\begin{array}{ccc} W(F,\Delta \mu ) & = & \frac{1}{2}NKT\left[\phi_{0}^{1/3}\left(I_{1}-3\right)-2\phi_{0}\ln J\right]\\ & & -\frac{KT}{v}\left[\begin{array}{c} \left(J-\phi_{0}\right)\ln\frac{J}{J-\phi_{0}}-\\ \chi \phi_{0}^{2}\frac{J-1}{J}+\left(1-\phi_{0}\right)\ln\left(1-\phi_{0}\right) \end{array}\right]\\ & & +\frac{\mu_{0}}{v}\left(1-J\right)-\frac{\Delta \mu }{v}\left(J-\phi_{0}\right), \end{array}$$

where $I_{1}=tr\left(F_{ik}F_{jk}\right)$, $J=\det(F_{ij})$, $\mu_{0}=Nv\phi_{0}^{1/3}+\chi \phi_{0}^{2}+\left(1-Nv\right)\phi_{0}+\ln\left(1-\phi_{0}\right)$, *v* is the volume of a water molecule, *K* is the Boltzmann constant, *T* is the temperature, Nv is a measure of the degree of cross-linking or cross-link density of the polymer network, and χ describes the interaction between the water molecule and polymer chain network (Flory–Huggins parameter). The quantity $\mu_{0}$ is the chemical potential at the reference state of initial swelling, and $\Delta \mu =\mu -\mu_{0}$ represents the change in chemical potential with respect to the reference state during swelling or de-swelling: swelling by the absorption of water occurs when $\Delta \mu /\mu_{0}<0$, while de-swelling occurs when $\Delta \mu /\mu_{0}>0$. The initial chemical potential $\mu_{0}$ is negative in the formula. Readers are referred to our recent work for more details [51]. The first Piola–Kirchhoff (PK) stress can be derived as

(2) $$\begin{array}{c} P_{ij}=\frac{\partial W}{\partial F_{ij}}=\frac{KT}{\upsilon }\left[Nv\phi_{0}^{1/3}F_{ij}+\left[\left(1-Nv\right)\frac{\phi_{0}}{J}-\frac{\mu_{0}+▵\mu }{KT}+\frac{\chi \phi_{0}^{2}}{J^{2}}+\ln\frac{J-\phi_{0}}{J}\right]J{\left(F^{-1}\right)}_{ji}\right]. \end{array}$$

The initial volume fraction of the polymer $\phi_{0}$ is a measurable quantity because the water content is known when the hydrogels are fabricated. However, Nv and χ are immeasurable and difficult to determine from the fabricated hydrogels; they need to be calibrated numerically by comparing with experiments, as detailed in the following. The commercial FE software ABAQUS (Version 6.10, Providence, RI, USA) was used [57] to simulate the surface instability of the bilayer hydrogel under constrained swelling. A user-defined material subroutine UHYPER (User subroutine to define a hyperelastic material) was employed to implement the proposed constitutive model.

The experimental compression tests were reproduced numerically by using the FE models of similar dimensions (cf. Figure 2b), loading, and boundary conditions. The FE models used to calibrate the material constants Nv and χ are given in Figure 2c. The mesh for the cylindrical sample is also given there. The element C3D8 (The 8-node brick element) was used in these simulations. The total number of elements was around 4000, following the mesh convergence study. It should be commented on here that Nv was hard to measure directly for the fabricated hydrogels. The proposed model was adopted with the undetermined parameter Nv to simulate the same block in the experiments. Nv was calibrated numerically by comparing the simulated force–displacement response with the experimental measured response, shown in the following.

Figure 2c shows the experimentally and numerically obtained force-engineering strain plots for three different volume fractions of water content. The experimental results with those from extensive FE simulations were fitted to calibrate Nv as $9\times 10^{-5}$, $5\times 10^{-5}$, and $5.5\times 10^{-6}$, respectively. The corresponding $\phi_{0}$ is also marked in Figure 2c. In the fitting of the experimental results, χ was taken to be 0.3, as is suggested by Milner and Lacasse [58], and χ ranged from 0.3 to 0.6 for the current samples.

Here we also assumed that the water content was almost homogeneous and that the water molecules did not diffuse during the compression test, setting Δμ=0. When a hydrogel is fabricated, the water molecules are in equilibrium with the fabrication environment. Thus the water content is almost homogeneous. When the specimen is taken out of the fabrication environment, the specimen is in the air. Depending on the humidity of the air, water molecules can diffuse into the specimen to be in equilibrium with the air environment. However, the diffusion of water molecules is much slower compared to the compressive loading. Thus it is only a very thin layer at the surface of specimen that can be inhomogeneous, which can be ignored for the stress–strain response. Then the assumption Δμ=0 is not perfect but is almost reasonable. While it is not shown here, our simulations also indicated that χ had insignificant influence on the force-engineering strain response for the hydrogel considered in this work (only the results of χ=0.3 are shown in the present paper). These simulations demonstrate that the proposed constitutive model can describe the mechanical behaviors of hydrogels well.

For the modeling of surface instability, the property of the hydrogel for other initial volume fractions of water should be set. It was impossible for us to carry out experiments on the specimens with every water volume fraction of the hydrogel. We thus set Nv in the material model as a function of the volume fraction of the polymer ($\phi_{0}$). We could fit Nv as a function of $\phi_{0}$ with the experimental measurements. This study found that the following formula could fit the experimental results very well, as shown in Figure 2d: $Nv=A\phi_{0}^{2}+B\phi_{0}+C$, where $A=4.6\times 10^{-3}$, $B=-2.96\times 10^{-5}$, and $C=-1.14\times 10^{-5}$. It is used for the instability analysis shown later.

### 3.2. Surface Instability Analysis

To fully understand the surface instability (creasing), the FE analysis was carried out through a two-step process: (1) buckling followed by (2) post-buckling analysis. Buckling analysis for finite-sized domains is described in the ABAQUS theory manual and previous works [31,33,38,59,60]. After determining the buckling modes from linear instability analysis, an imperfection in the form of the most critical eigenmode was introduced into the initial FE mesh. The mesh was perturbed by the corresponding eigenmode and scaled by a factor ω. It was shown by [31] that the surface morphology is imperfection-sensitive. The perturbation cannot be too large because the FE mesh deviates from the initial model. It also cannot be too small because it may not reach the instability pattern. Our results demonstrate that when the imperfection was large enough (ranged from 0.003 to 0.006), the results were the same for these simulations. We show only the results of w=0.003H with ω=0.003 here.

In addition to buckling and post-buckling FE analyses, a theoretical linear instability analysis was also carried out by the method proposed by Tang et al. [15]. The proposed method by Tang et al. [15] is designed for neo-Hookean solids. If we replace the first PK stress and tangent modulus for the neo-Hookean solids by Equation (2) and

(3) $$\begin{array}{ccc} B_{ijkl}^{0} & = & \frac{\partial P_{ij}}{\partial F_{kl}}=\frac{NvKT}{\upsilon }\phi_{0}^{1/3}\delta_{ik}\delta_{jl}+\\ & & \frac{1}{2}\frac{KT}{\upsilon }\left(\begin{array}{c} -\frac{\mu_{0}+▵\mu }{KT}J+\frac{\chi \phi_{0}^{2}}{J}+J\ln\frac{J-\phi_{0}}{J}\\ -2\frac{\chi \phi_{0}^{2}}{J}+\frac{J^{2}}{J-\phi_{0}}-J \end{array}\right){\left(F^{-1}\right)}_{ji}\left(C_{ml}^{-1}F_{km}+C_{lm}^{-1}F_{km}\right)-\\ & & \frac{KT}{\upsilon }\left[\left(1-N_{v}\right)\frac{\phi_{0}}{J}-\frac{\mu_{0}+▵\mu }{KT}+\frac{\chi \phi_{0}^{2}}{J^{2}}+\ln\frac{J-\phi_{0}}{J}\right]J{\left(F^{-1}\right)}_{jk}{\left(F^{-1}\right)}_{li} \end{array}$$

for the proposed constitutive model of the hydrogel, the critical compressive strain and the corresponding wave modes can be obtained using the same approach.

## 4. Results and Discussion

**Figure 3:**
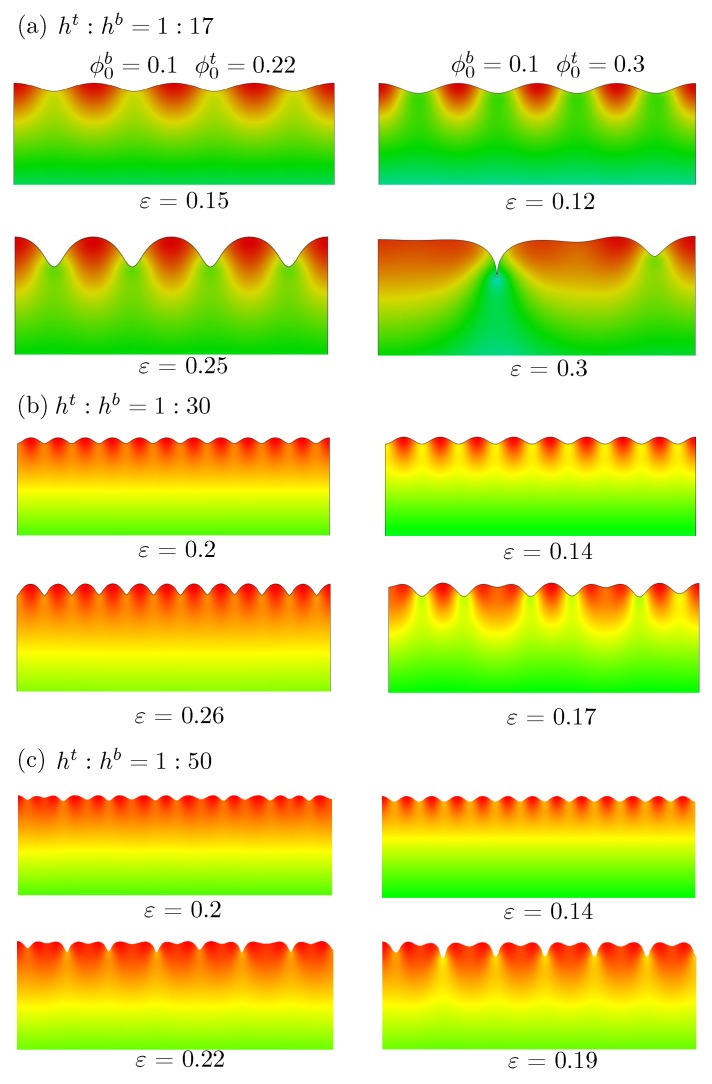
Surface morphologies of bilayer hydrogels under different levels of compressive strain marked below each figure for different thickness ratios of upper and lower layers: (**a**) $h^{t}/h^{b}=1/17$; (**b**) $h^{t}/h^{b}=1/30$; (**c**) $h^{t}/h^{b}=1/50$. The initial volume fraction of polymer chain in the lower layer was $\phi_{0}^{b}=0.1$. Two initial volume fractions of polymer chain in the upper layer were considered: $\phi_{0}^{b}=0.22$ and $\phi_{0}^{b}=0.3$, shown in the left and right columns, respectively.

This study first shows the effect of the upper-layer thickness through FE simulations. The displacement in the *y*-direction of the bottom edge and the left boundary in the *x*-direction was fixed. The uniform displacement in the *x*-direction was set as zero in the expansion stage and imposed monotonically on the right edge in the compression process. Figure 3 shows deformation configurations for three different thickness ratios $h^{t}/h^{b}$ of 1/17, 1/30, and 1/50 under plane strain conditions at different levels of compression in our post-buckling analysis. Here, the superscripts *t* and *b* represent the upper and lower layers, respectively. The compressive strain is marked below each figure. These strain levels were chosen because the typical instability patterns are shown clearly. The initial volume fraction of the polymer for the bottom layer was $\phi_{0}^{b}=0.1$, while the top layer was $\phi_{0}^{t}=0.22$ or 0.3. When $h^{t}/h^{b}=1/50$, the thickness of the top layer was much smaller compared with the substrate, which was consistent with the bilayer system studied by many previous researchers [31,46]. For both cases ($\phi_{0}^{b}=0.1,\phi_{0}^{t}=0.22$) and ($\phi_{0}^{b}=0.1,\phi_{0}^{t}=0.3$), we can see that as the compressive deformation increased, wrinkles formed on the flat surface first and evolved into the pattern of period doublings later. This period doubling was first reported by compression on the bilayer structure of the hard thin film (PDMS) on soft substrate (PDMS) through experiments [46]. Later, the FE simulations by Cao and Hutchinson [31] further confirmed this interesting phenomenon. Their work also shows that when the modulus ratio between the thin film and substrate is greater than 5, the wrinkles change into period doubling (see Figure 9 therein). When the modulus ratio between the thin film and substrate is less than 5, the initial wrinkles transform into a local fold and creasing (see Figure 10 therein). When the thickness of the thin film was larger ($h^{t}/h^{b}=1/30$), we observed that the wrinkles appeared first but periodic doubling could not be observed for the ($\phi_{0}^{b}=0.1,\phi_{0}^{t}=0.22$) case, even when the imposed compressive strain was around 26%. For ($\phi_{0}^{b}=0.1,\phi_{0}^{t}=0.3$), the wrinkles transformed into tripling. Such tripling is reported by Auguste et al. [49] for a soft bilayer with a pre-stretched substrate. When the thickness of the thin film is much larger ($h^{t}/h^{b}=1/17$), we can see that wrinkles appeared first but periodic doubling could not be observed for ($\phi_{0}^{b}=0.1,\phi_{0}^{t}=0.22$). For ($\phi_{0}^{b}=0.1,\phi_{0}^{t}=0.3$), the tripling did not appear, but folds were observed.

Summarizing the results in Figure 3, we can conclude that when the thickness of the upper layer is increasing and is a little stiffer than the substrate ($\phi_{0}^{t}$ is a little greater than $\phi_{0}^{b}$), the periodic doublings cannot appear and only surface wrinkles can exist. When the thickness of the upper layer is increasing and is much stiffer than the substrate ($\phi_{0}^{t}$ is much larger than $\phi_{0}^{b}$), periodic doubling can evolve into folds. In fact, the theoretical prediction given by Brau et al. [46] on the basis of bilayer PDMS/PDMS experimental data shows that the critical compression is not related to the thickness of the upper layer ($\epsilon^{crit}=0.02{\left(1-\nu \right)}^{2}/{\left(1-2\nu \right)}^{2}$, where ν is Poisson’s ratio), because their work assumed that the top layer was very thin compared with the substrate and plate theory was employed to describe the deformation of the upper layer.

**Figure 4:**
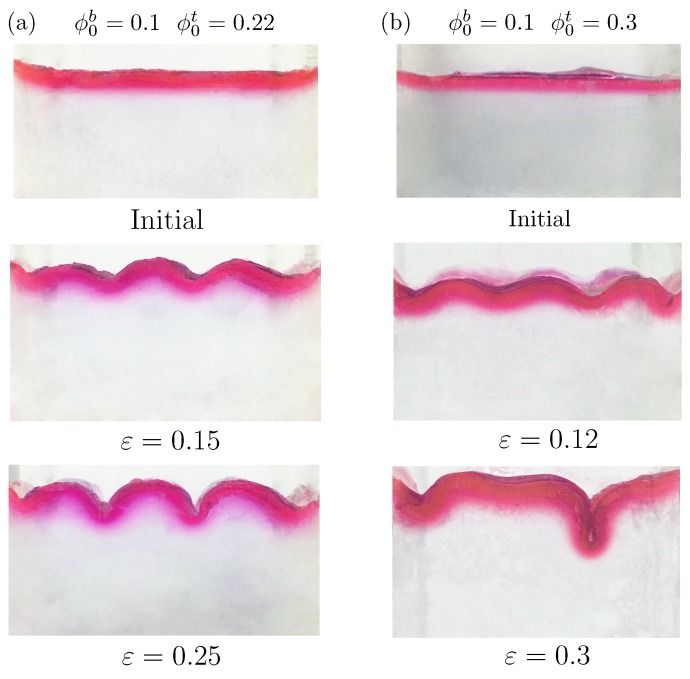
Surface morphologies of bilayer hydrogels under different levels of compressive strain for the thickness ratio of the upper and lower layers $h^{t}/h^{b}=1/17$ in the experiments. The compressive strain is marked below each figure. The initial volume fraction of polymer chain in the lower layer is $\phi_{0}^{b}=0.1$. Two initial volume fractions of polymer chain in the upper layer are considered: (**a**) $\phi_{0}^{t}=0.22$; (**b**) $\phi_{0}^{t}=0.3$.

This study thus shows the experimental results to confirm the above simulation results. A bilayer hydrogel was fabricated with $\phi_{0}^{b}=0.1$ and $\phi_{0}^{t}=0.22$ or 0.3. The thickness ratio between the top layer and bottom layer was 1/17. Figure 4 shows the deformation configurations under different levels of compressive strain in the experiments. The cases ($\phi_{0}^{b}=0.1$, $\phi_{0}^{t}=0.22$) and ($\phi_{0}^{b}=0.1$, $\phi_{0}^{t}=0.3$) are shown in Figure 4a,b, respectively. For the case ($\phi_{0}^{b}=0.1$, $\phi_{0}^{t}=0.22$), only wrinkles on the surface were observed, consistent with our numerical predictions (cf. Figure 3a). Further increments in the compressive strain could cause fracture of the hydrogel. For the case ($\phi_{0}^{b}=0.1$, $\phi_{0}^{t}=0.3$), the wrinkles transformed into folds directly without periodic doubling. The experimental observations showed good agreement with the simulation results given in Figure 3. The experiments also demonstrated the effectiveness of the proposed constitutive law for this hydrogel from another aspect.

**Figure 5:**
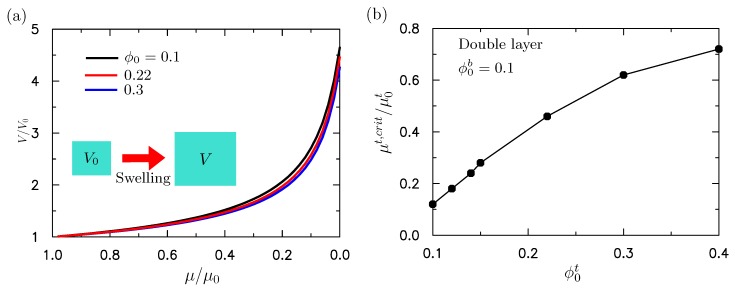
(**a**) Volume expansion $V/V_{0}$ vs $\mu /\mu_{0}$ predicted by the proposed constitutive model for hydrogels. The decrease in $\mu /\mu_{0}$ led to the volume expansion of hydrogels. (**b**) The critical volume expansion ($\mu^{t,crit}/\mu_{0}^{t}$) at which the surface instability occurred as a function of the initial volume fraction of the polymer chain $\phi_{0}^{t}$. The initial volume fraction of the polymer chain of the lower layer was fixed as $\phi_{0}^{b}=0.1$. The surface instability was purely induced by the absorption of water in the upper layer.

The surface instability of a bilayered structure can be affected by the absorption of water in the top layer, the mechanical compression, or both. The surface instability under the pure mechanical compression has been discussed in the above sections. We now discuss the absorption of water and the combined absorption of water and mechanical compression on the surface instability of bilayer hydrogels. We studied the influence of water absorption on the surface instability first through theoretical analysis. This study focused on the conventional thin film/substrate system. Then we set $h^{t}/h^{b}=1/50$, as described in the following discussions. As discussed in our constitutive model, the swelling by absorption of water occurred when $\Delta \mu /\mu_{0}<0$, while de-swelling occurred when $\Delta \mu /\mu_{0}>0$. This was further demonstrated by a unit cell with initial volume $V_{0}$ and initial chemical potential $\mu_{0}$. When we reduced the chemical potential from the initial $\mu_{0}$ value in the simulation, it was found that the volume of the unit cell increased. The results are presented in Figure 5a for the hydrogel with initial volume fractions of the polymer of $\phi_{0}=0.1$, 0.22, 0.3.

**Figure 6:**
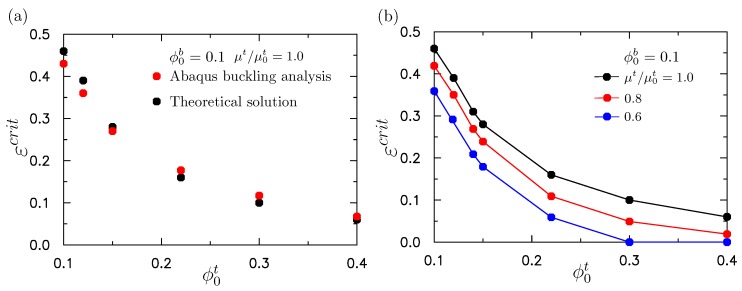
(**a**) Comparison of predictions for the critical strain at which the surface instability occurs for bilayer structure as a function of the initial volume fraction of polymer chain in the upper layer by the finite element (FE) simulation and theoretical analysis. There is no absorption of water in the upper layer with $\mu^{t}/\mu_{0}^{t}=1$. (**b**) The critical compressive strain at which the surface instability occurred as a function of the initial volume fraction of polymer chain in the upper layer under different levels of the pre-absorption of water: $\mu^{t}/\mu_{0}^{t}=1$, 0.8, 0.6. The initial volume fraction of polymer chain of the lower layer was fixed as $\phi_{0}^{b}=0.1$.

To mimic the absorption of water in the upper layer, the chemical potentials of the upper layer is decreased and the initial volume fraction of polymer chain in the bottom layer is fixed as $\phi_{0}^{b}=0.1$. When the chemical potential was reduced to a critical value, the compressive stress induced by the expansion of the gel could lead to surface instability. Figure 5b shows the critical chemical potential versus the initial volume fraction of polymer chains $\phi_{0}^{t}$ in the top layer. We can see from Figure 5b that the chemical potential required to cause the surface instability decreased with the initial volume fraction of the polymer $\phi_{0}^{t}$. When $\phi_{0}^{t}$ was larger than $\phi_{0}^{b}$, this indicated that the modulus of the upper layer was larger than that of the lower layer. As shown in the previous analysis on bilayer neo-Hookean solids [31], a larger ratio of the modulus leads to a smaller compressive strain required to trigger the surface instability. Similarly, when $\phi_{0}^{t}$ is much larger than $\phi_{0}^{b}$, the compressive stress required to trigger the surface instability is smaller. Then the required reduction in chemical potential is smaller. That is, $\mu /\mu_{0}$ is larger. The results shown in Figure 5 are consistent with the trends given by Cao and Hutchinson [31]. This study thus shows the effect of the combined absorption of water and mechanical compression by theoretical analysis. It first shows the accuracy of the theoretical analysis by comparison with the buckling analysis through ABAQUS. The predicted critical strain when the surface instability occurs as a function of the initial volume fraction of the polymer chain is shown in Figure 6a without the initial absorption of the water ($\mu^{t}/\mu_{0}^{t}=1$). We can see from Figure 6a that the prediction given by the theoretical analysis is in good agreement with the ABAQUS buckling analysis.

The critical compressive strains at which the surface instability occurred as a function of the initial volume fraction of the polymer chains under three levels of initial absorption of water ($\mu^{t}/\mu_{0}^{t}=1$, 0.8, 0.6) are shown in Figure 6b. Here, the absorption of water in the upper layer before the compression was considered. It can be seen that the critical compressive strain decreased with increasing $\phi_{0}^{t}$. If the initial absorption of water was larger ($\mu^{t}/\mu_{0}^{t}$ was smaller), the required compressive strain leading to the surface instability was smaller, because greater absorption of the water resulted in a larger initial compressive stress. The results shown in Figure 6 are consistent with our intuition.

**Figure 7:**
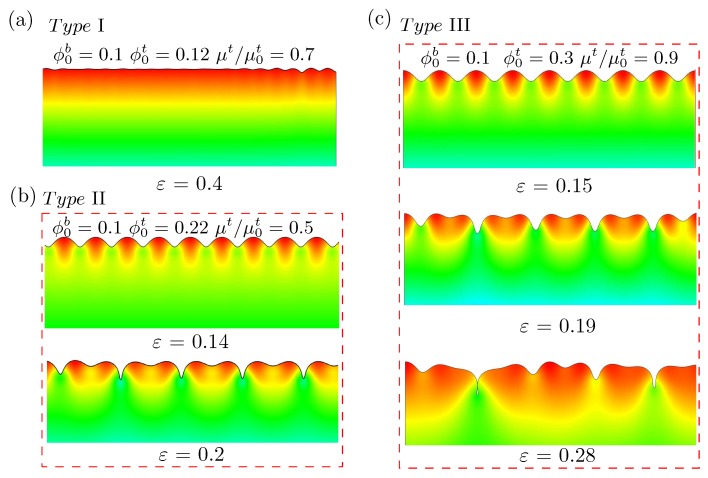
Surface morphologies of bilayer hydrogel modeled by the finite element (FE) simulations under different levels of compressive strain with the pre-absorption of water in the upper layer, when the initial volume fraction of polymer chain $\phi_{0}^{t}$ varied and the lower layer was fixed as $\phi_{0}^{b}=0.1$: (**a**) $\phi_{0}^{t}=0.12$, (**b**) $\phi_{0}^{t}=0.22$, and (**c**) $\phi_{0}^{t}=0.3$. These are typical surface instability patterns, named types I, II, and III, respectively.

The post-buckling analysis on bilayer hydrogels through ABAQUS was performed and is given in this section. Similarly to that shown in Figure 6, the top layer absorbed water first, followed by the mechanical compression. This study shows the deformation configuration at a compressive strain of 0.4 for ($\phi_{0}^{b}=0.1$, $\phi_{0}^{t}=0.12$) and $\mu^{t}/\mu_{0}^{t}=0.7$ in Figure 7a. In this case, the wrinkles with small amplitude transformed into multiple localizations. With the slight increment in compression, the localization continued to develop into the form of an incipient fold. This configuration was very similar to that shown in Figure 10 of [31] for bilayer neo-Hookean solids with a modulus ratio between the top and bottom layers of less than 10.

In Figure 7b, the deformation configurations at compressive strains of 0.14 and 0.2 for the ($\phi_{0}^{b}=0.1$, $\phi_{0}^{t}=0.22$) and $\mu^{t}/\mu_{0}^{t}=0.5$ cases are shown. We can see in Figure 7b that the sinusoidal wrinkles were stable prior to the onset of period doublings. This configuration was very similar to that shown in Figure 9 of [31] for bilayer neo-Hookean solids with a modulus ratio between the upper and lower layers of greater than 10. The present work shows that these two behaviors are also representative of bilayer hydrogels.

When we further increased the volume fraction of polymer chains in the top layer, ($\phi_{0}^{b}=0.1$, $\phi_{0}^{t}=0.3$) and $\mu^{t}/\mu_{0}^{t}=0.9$, the deformation configurations were as presented in Figure 7c. It can be seen that wrinkles evolved into period doublings and transformed into folds at larger compressive deformations. This behavior was qualitatively different from what is given in [31] for bilayer neo-Hookean solids. However, it is similar to Figure 12 shown in [31] with a small pre-stretch in the complainant substrate. This demonstrates the importance of the initial absorption of water in the upper layer on the surface instability of bilayer hydrogels.

**Figure 8:**
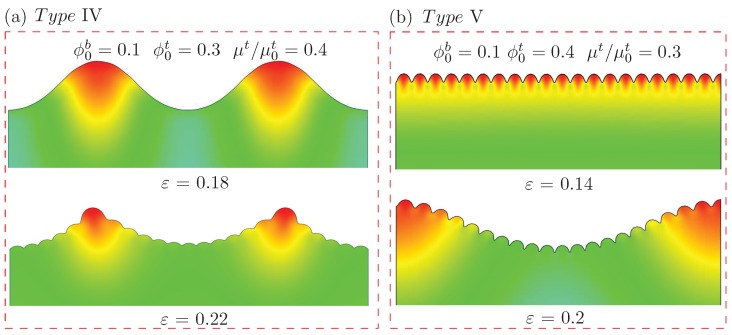
Surface morphologies of bilayer hydrogel modeled by the finite element (FE) simulations under different levels of compressive strain with the pre-absorption of water in the upper layer, when the initial volume fraction of polymer chain in the bottom layer was fixed as $\phi_{0}^{b}=0.1$. The initial volume fraction of polymer chain in the upper layer $\phi_{0}^{t}$ was much larger with (**a**) $\phi_{0}^{t}=0.3$, and (**b**) 0.4. These are typical surface instability patterns, named types IV and V, respectively.

When the volume fraction of the polymer chains in the upper layer was much larger than that in the lower layer, with more absorption of water in the upper layer, the representative deformation configurations under different imposed compressive strains were as shown in Figure 8a,b. The material constants were $\phi_{0}^{b}=0.1$ and $\phi_{0}^{t}=0.3$ with the pre-absorption of water characterized by $\mu^{t}/\mu_{0}^{t}=0.4$ for Figure 8a, while they were $\phi_{0}^{b}=0.1$ and $\phi_{0}^{t}=0.4$ with $\mu^{t}/\mu_{0}^{t}=0.3$ for Figure 8b. In the case shown in Figure 8a, wrinkles with a large wavelength appeared first. With the increment in compressive deformation, the appearance of secondary wrinkles with a smaller wavelength on the wrinkles with a large wavelength led to a hierarchical wavy structure. To the best of our knowledge, there have been no reported experimental observations of hierarchical wrinkles in bilayer hydrogels. However, different approaches for other soft material systems such as PDMS are proposed [50,61]. Shao et al. [50] used the surface wrinkles to fabricate a surface with periodic variation in curvature. The formation of a hierarchical wrinkling pattern could be controlled by regulating the curvature of the surface. Wang et al. [62] recently proposed an approach to utilize a three-layer system (an aluminum thin film deposited on a hard PDMS, attached to a pre-stretched soft PDMS substrate) to form a wrinkling pattern with three orders. In the case shown in Figure 8b, the wrinkles with a small wavelength appeared first. With the further increment in compressive strain, wrinkles with a larger wavelength occurred. These also formed a hierarchical pattern of wrinkles on the surface.

**Figure 9:**
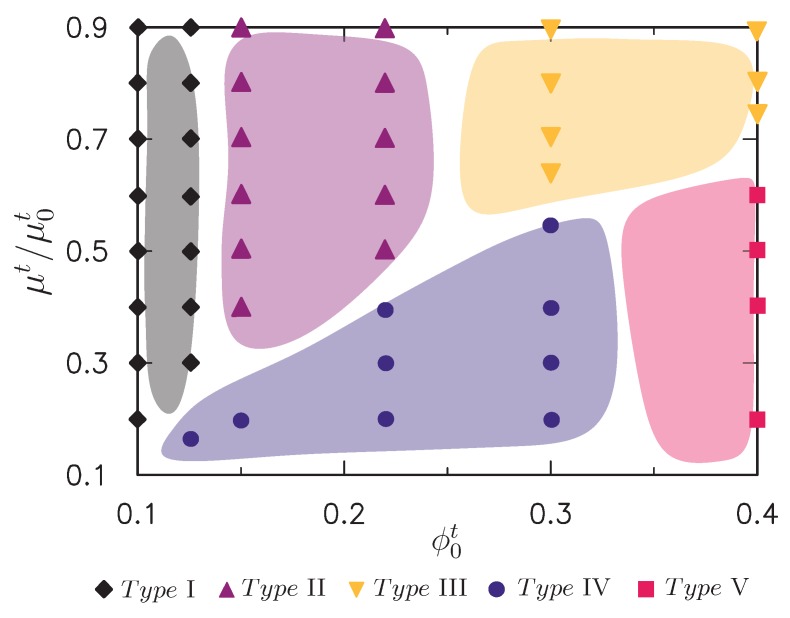
Predicted phase diagram by finite element (FE) simulations for the occurrence of typical surface morphologies (types I, II, III, IV, and V) shown in Figure 7 and Figure 8. The phase diagram was constructed as a function of the initial absorption of water $\mu^{t}/\mu_{0}^{t}$ and initial volume fraction of polymer chain $\phi_{0}^{t}$ of the bilayer hydrogel under both the pre-absorption of water in the upper layer and mechanical compression. The initial volume fraction of polymer chain in the bottom layer was fixed as $\phi_{0}^{b}=0.1$.

According to the above computational observations, extensive FE simulations were performed for wide ranges of the initial absorption of water value ($\mu^{t}/\mu_{0}^{t}$) and initial volume fraction of the polymer chains in the top layer ($\phi_{0}^{t}$). The initial volume fraction of the polymer chains in the bottom layer was fixed as $\phi_{0}^{b}=0.1$. Figure 9 presents our numerical findings in a form analogous to a phase diagram, wherein the abscissa denotes $\phi_{0}^{t}$ and the ordinate is $\mu^{t}/\mu_{0}^{t}$. The discrete points shown correspond to the discrete levels of $\mu^{t}/\mu_{0}^{t}$ and $\phi_{0}^{t}$ analyzed, where black filled quadrangles, purple filled triangles, and yellow filled inverted triangles represent the formation of surface patterns of types I, II, and III, respectively, in Figure 7. Blue full circles and red full squares in Figure 8 denote types IV and V, respectively. With this phase diagram at hand, it was possible for us to tune the absorption of the water and initial volume fraction of polymer chains of the upper layer to control the surface morphology.

## 5. Conclusions

Through theoretical analyses, FE simulations, and experiments, this study explored the effects of the thickness and pre-absorption of water in the upper layer on the surface instability of a bilayer hydrogel under compression. This study found that the period doublings on the bilayer hydrogel can be suppressed when the thickness of the upper layer is larger. The surface instability behaviors of the bilayer system can be greatly affected by the thickness ratio between the upper and lower layers. In addition to the local folds and period doublings observed for neo-Hookean bilayer structures in previous studies [31], the transformation from period doublings to folds and hierarchical wrinkles was also observed for bilayer hydrogels by the initial absorption of the water followed by compression. Thus, both the thickness of the substrate and absorption of water molecules can be used to tune the surface instability patterns of bilayer hydrogels under compression. Further experimental works are ongoing and we are aiming to reduce the thickness of the upper layer of the bilayer hydrogel for further experiments. With further efforts, we believe that all the surface patterns reported in this work will be demonstrated fully by experiments.
